# SNP discovery and genetic structure in blue mussel species using low coverage sequencing and a medium density 60 K SNP‐array

**DOI:** 10.1111/eva.13552

**Published:** 2023-04-25

**Authors:** Jennifer C. Nascimento‐Schulze, Tim P. Bean, Carolina Peñaloza, Josephine R. Paris, James R. Whiting, Alexis Simon, Bonnie A. Fraser, Ross D. Houston, Nicolas Bierne, Robert P. Ellis

**Affiliations:** ^1^ Biosciences, Faculty of Health and Life Sciences University of Exeter Exeter UK; ^2^ Centre for Environment, Fisheries and Aquaculture Science Weymouth Laboratory Weymouth UK; ^3^ The Roslin Institute and Royal (Dick) School of Veterinary Studies University of Edinburgh Midlothian UK; ^4^ ISEM University of Montpellier, CNRS, IRD Montpellier France; ^5^ Benchmark Genetics Midlothian UK; ^6^ Centre for Sustainable Aquaculture Futures University of Exeter Exeter UK

**Keywords:** aquaculture, mussels, *Mytilus*, population genomics, SNP, SNP chip

## Abstract

Blue mussels from the genus *Mytilus* are an abundant component of the benthic community, found in the high latitude habitats. These foundation species are relevant to the aquaculture industry, with over 2 million tonnes produced globally each year. Mussels withstand a wide range of environmental conditions and species from the *Mytilus edulis* complex readily hybridize in regions where their distributions overlap. Significant effort has been made to investigate the consequences of environmental stress on mussel physiology, reproductive isolation, and local adaptation. Yet our understanding on the genomic mechanisms underlying such processes remains limited. In this study, we developed a multi species medium‐density 60 K SNP‐array including four species of the *Mytilus* genus. SNPs included in the platform were called from 138 mussels from 23 globally distributed mussel populations, sequenced using a whole‐genome low coverage approach. The array contains polymorphic SNPs which capture the genetic diversity present in mussel populations thriving across a gradient of environmental conditions (~59 K SNPs) and a set of published and validated SNPs informative for species identification and for diagnosis of transmissible cancer (610 SNPs). The array will allow the consistent genotyping of individuals, facilitating the investigation of ecological and evolutionary processes in these taxa. The applications of this array extend to shellfish aquaculture, contributing to the optimization of this industry via genomic selection of blue mussels, parentage assignment, inbreeding assessment and traceability. Further applications such as genome wide association studies (GWAS) for key production traits and those related to environmental resilience are especially relevant to safeguard aquaculture production under climate change.

## 1 BACKGROUND

Blue mussels from the genus *Mytilus* are an abundant component of the benthos, found in high latitude habitats (Gosling, 2015). These foundation species can aggregate in high densities, forming extensive beds or reefs, which provide a number of important ecosystem services (e.g. providing spatial structure, undertaking nutrient cycling, and forming an important food source) (van der Schatte Olivier et al., 2020). Additionally, mussels play an important economic role, as both a fishery and aquaculture species, accounting for approximately 12%, or ~2 million tonnes, of global mollusc production (Subasinghe, 2017). Most landings (>90%) derive from aquaculture (Avdelas et al., 2021), with farmed bivalves identified as one of the most sustainable sources of animal protein (Hilborn et al., 2018). From a nutritional perspective, mussels contain high levels of omega‐3 polyunsaturated fatty acids and essential amino acids, which in the human diet have significant health benefits (Carboni et al., 2019). Commercial blue mussel production in Europe relies almost exclusively on collection of naturally‐settled spat (i.e. settled juveniles) (Kamermans et al., 2013). Several environmental factors (e.g. water temperature, salinity, food availability, and local currents) influence the reproductive cycle, in addition to triggering spawning events and determining larval dispersal patterns.

Species from the *M. edulis* species complex (*M. edulis*, *M. trossulus*, and *M. galloprovincalis*), the main blue mussel species prevailing in the northern hemisphere, and their southern counterpart *M. chilensis*, are the *Mytilus* species with the highest economic value in terms of aquaculture production. They are also the species that, wherever their geographic range overlaps, readily hybridize. This includes regions occurring in the south west of the United Kingdom (Gardner, 1996; Hilbish et al., 2002; Vendrami et al., 2020), the European coast of the north east Atlantic (Bierne et al., 2002; Bierne, Borsa, et al., 2003; Fraïsse et al., 2016; Simon et al., 2021), north west Atlantic (Koehn et al., 1984; Rawson et al., 2001; Toro et al., 2004), the Baltic sea (Riginos & Cunningham, 2005; Stuckas et al., 2017; Väinölä & Hvilsom, 1991), subarctic and arctic regions (Mathiesen et al., 2017), the north east Pacific (Rawson et al., 1999; Saarman & Pogson, 2015), south and east Pacific (Larraín et al., 2019; Popovic et al., 2020). Nonetheless, in the Northern hemisphere *M. californianus* and *M. coruscus* also coexist with the previously mentioned taxa, but do not seem to readily hybridize. In the Southern hemisphere *M. chilensis* coexists with other distinct lineages of blue mussels including *M. platensis*, *M. aoteanus* from New Zealand and *M. planulatus* from Australia, together with the recently introduced Northern hemisphere *M. galloprovincialis* (Oyarzún et al., 2021; Popovic et al., 2020; Zbawicka et al., 2018). Broad‐scale population structure and population dynamics in this species complex is therefore predominantly shaped by interactions between oceanography and the biology of each species. Both pre‐ and post‐settlement selection drive geographical and ecological segmentation, and contribute to determining species distribution and in shaping hybrid zones (Bierne et al., 2002; Bierne, Bonhomme, & David, 2003; Knöbel et al., 2021; Koehn et al., 1980). Moreover, the success of mussel aquaculture is tightly coupled to the environment, across all stages of production, with environmental change also influencing key performance traits including growth, survival, and susceptibility to disease (Nascimento‐Schulze et al., 2021). Therefore, further research is needed to fully understand the genetic barriers that determine distribution of these taxa, as well as the genomic mechanisms driving such processes across different environments.

Selective breeding has been highlighted as a key tool to facilitate sustainable intensification of bivalve aquaculture, allowing the development of specialized breeding lines resilient to environmental and pathogenic challenges (FAO, 2016; Nascimento‐Schulze et al., 2021; Potts et al., 2021). Selection has benefited the production of many cultured aquatic taxa (Gjedrem & Rye, 2018), including the most important marine bivalve molluscs such as mussels and oysters (Hollenbeck & Johnston, 2018). Methods of selection vary from mass selection, for example breeding from the fastest growers in a population, to methods using genetic markers spread across the genome, known as genomic selection (GS). GS is particularly powerful as it can improve accuracy of selective breeding, contributing to highly targeted results, even in the case of polygenic traits, whilst allowing for full control of the genetic relationships of the offspring (Houston et al., 2020; Meuwissen et al., 2001). Nonetheless, GS approaches in marine bivalves are still in their infancy, and their efficacy within different species require significant investigation to be fully elucidated. The development of a SNP‐array for blue mussels will therefore contribute to the investigation of whether GS is a viable option to enhance aquaculture production in these taxa.

To utilize GS, genome‐wide markers are required. In most organisms, single nucleotide polymorphisms (SNPs) are the most common form of genetic variation and, therefore, the marker of choice for GS. Other forms of structural genetic variation, including large insertions and deletions, are known to be prevalent in blue mussels (Gerdol et al., 2020), and therefore may play an important role determining phenotypic diversity in this group. However, SNPs occur in genes/regulatory regions, as well as non‐coding regions and still provide relevant information on phenotype and trait heritability. Whilst these markers may be contributing to the expressed phenotype, using a high density of markers can assure that a majority are in linkage disequilibrium (LD) with causal mutations. Advances in sequencing and computational technologies have facilitated lowering costs of SNP discovery and enable the generation of large quantities of sequencing data and high throughput screening of SNPs. Consequently, these genetic markers have been widely applied for the development of further genomic resources.

SNP‐arrays use a probe‐based approach to generate high‐quality genotype data whilst requiring less investment in sample preparation. Computational analysis of genotype data generated by arrays is less demanding than techniques such as genotyping‐by‐sequencing that provide a similar amount of data. Furthermore, SNP‐arrays enable genotyping in multiple pre‐defined loci, guaranteeing reproducibility of analysis (Robledo et al., 2018). Such information contributes to our understanding of genomic processes underpinning research in evolutionary genomics, population genetics, conservation and ecology (Allendorf et al., 2010; Andrews et al., 2016). Therefore, SNP‐arrays have valuable applications for the study of both wild and farmed populations and have been successfully applied across multiple taxa (Houston et al., 2014; Kranis et al., 2013; Michelizzi et al., 2011; Stoffel et al., 2012). Arrays are currently available for several farmed aquatic species including the Pacific (*Crassostrea gigas*) and European flat oysters (*Ostrea edulis*) (Gutierrez et al., 2017; Lapègue et al., 2014; Qi et al., 2017), Atlantic salmon (*Salmo sala*r) (Houston et al., 2014), Rainbow trout (*Onchorynchus mykiss*) (Bernard et al., 2022; Palti et al., 2015) and Nile Tilapia (*Oreochromis niloticus*) (Joshi et al., 2018; Peñaloza et al., 2020), offering a rapid, accessible and cost‐effective approach for medium‐ and high‐density genotyping.

In blue mussels, nuclear and mitochondrial DNA markers have been widely used to investigate evolutionary processes (Quesada et al., 1998; Rawson & Hilbish, 1995; Zouros et al., 1994), species genetics (Koehn, 1991; McDonald et al., 1991), hybridization patterns (Bierne, Borsa, et al., 2003; Riginos & Cunningham, 2005; Stuckas et al., 2017) and selection (Bierne, Bonhomme, & David, 2003; Fraïsse et al., 2016; Knöbel et al., 2021; Koehn et al., 1980). Linkage maps have been developed for *Mytilys edulis* (Lallias et al., 2007), whilst low‐density SNP‐panels have been used to delineate species and genetic lineages within the *Mytilus* genus (Simon et al., 2021; Wilson et al., 2018), to investigate the shell trait‐species correlation (Carboni et al., 2021) and also for pedigree reconstruction (Nguyen et al., 2014a). Studies have also applied different genotyping methods including low‐density panels (Gardner et al., 2016; Saarman & Pogson, 2015; Wenne et al., 2016; Zbawicka et al., 2014, 2018), as well as next generation sequencing (NGS), to investigate genetic diversity structure in *Mytilus* populations across the globe (e.g. Fraïsse et al., 2016; Nguyen et al., 2014b; Vendrami et al., 2020), which has led to the development of an 81‐SNP Fluidigm genotyping assay panel (Mathiesen et al., 2017) and a 212‐SNP ancestry informative panel (Simon et al., 2021). Finally, the recently assembled genomes of *M. edulis*, *M. galloprovincialis*, *M. coruscus*, *M. chilensis and M. californianus* (Corrochano‐Fraile et al., 2022; Gallardo‐Escárate et al., 2022; Gerdol et al., 2020; Paggeot et al., 2022; Simon, 2022; Yang et al., 2021), will undoubtedly facilitate the development of inclusive and genome‐wide tools for these taxa. Low density marker panels are underpowered for many important questions, including GWAS to identify polygenic QTLs, population structure and speciation in these taxa. High density panels can resolve such open questions, consequently fast‐tracking genetic improvement in mussels.

In this study, we generate a SNP database for the *Mytilus edulis* complex (*M. edulis*, *M. galloprovincialis* and *M. trossulus)*, together with the southern hemisphere, *Mytilus chilensis*, given the high economic relevance of these species to the mussel industry, as well as the ecological significance of this group given their propensity to hybridize. A subset of this database comprising 60 K SNPs was then used to develop a medium density SNP‐array on the Affymetrix ThermoFisher platform. Polymorphic markers were discovered using low‐coverage whole‐genome sequencing (lcWGS) data of 23 globally distributed blue‐mussel populations, including multiple pure and hybrid genotypes. This tool will facilitate the investigation of population genetic processes such as local adaptation and reproductive isolation in this species complex, and increase the potential of selective breeding in mussel aquaculture.

## 2 MATERIALS AND METHODS

### 2.1 Sample collection, DNA extraction and sequencing

**FIGURE 1:**
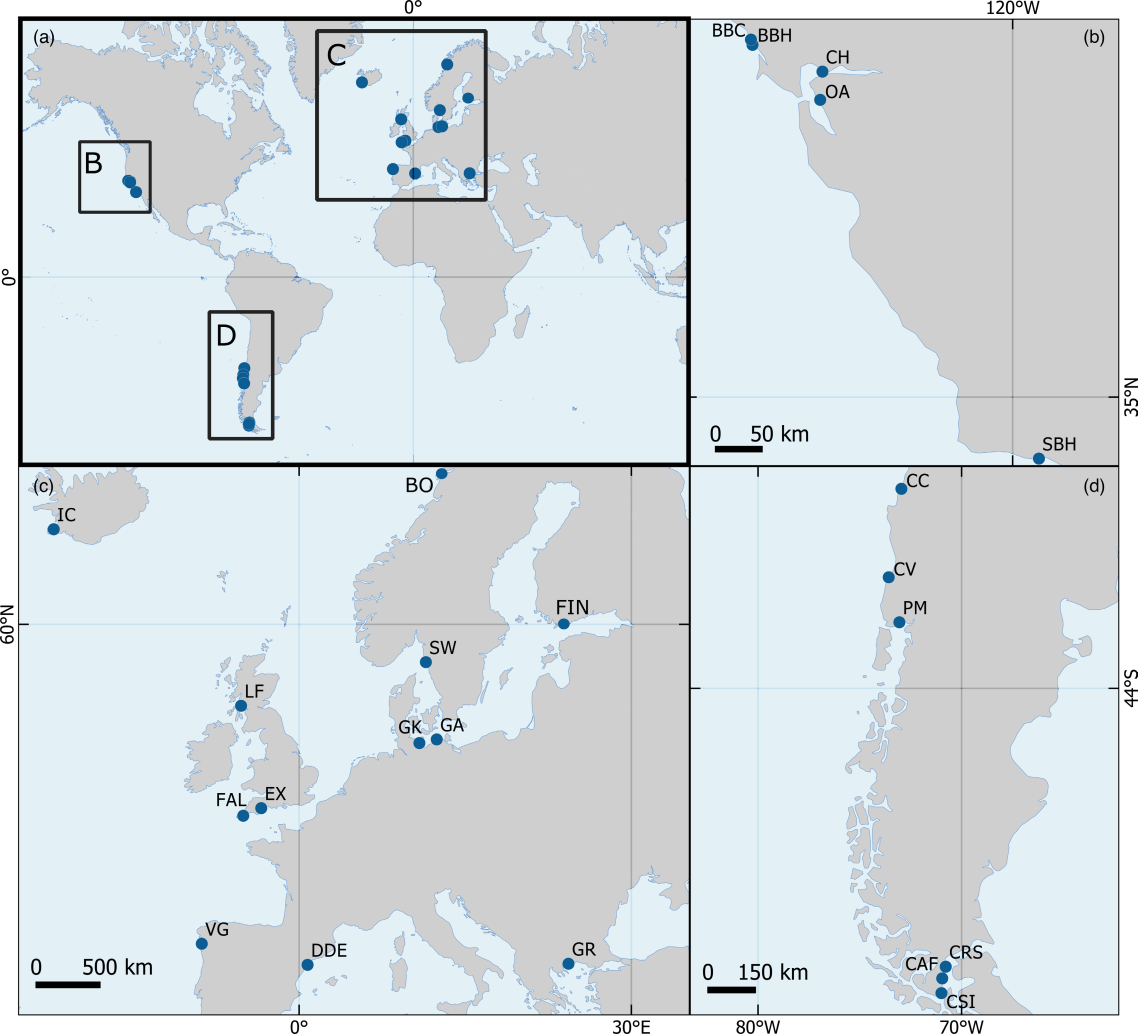
Global distribution (a) of the 23 Blue mussel populations used in this study to generate WGS data. Segments (b), (c), and (d) of the figure detail the location of populations in the North East Pacific, North East Atlantic, Baltic,and Mediterranean Seas and South East Pacific, respectively.

Twenty‐three *Mytilus* spp populations were sampled from across their global distribution in 2018 (Figure 1, Table 1), incorporating three species in this complex (*M. edulis*, *M. trossulus and M. galloprovincialis)* and their hybrids, together with the southern *M. chilensis*. DNA was extracted from ethanol (96%) preserved adductor muscle tissue using the E.Z.N.A.® mollusc DNA kit (Omega Bio‐Tek). Extraction followed the manufacturers protocol except the following adjustment: 30 mg of tissue was first homogenized (FastPrep‐24™ 5G, MP Biomedicals™) for 1 min (30 + 30 s) at 6 m s^−1^ in 350 mL of ML1 buffer, and subsequently digested for 1–4 h at 50°C (until no tissue was visible in the vials) with 25 μL of proteinase K, prior to extraction. DNA was assessed using a NanoDrop One Spectrophotometer (A260/280 and 260/230 ratios) and a Qubit BR assay. Whole‐genome sequencing (WGS) library preparation and sequencing of six individuals per population (*n* = 138) was performed by the University of Exeter Sequencing Service. Libraries were prepared with the NEBNext Ultra II FS DNA Sample Preparation Kit (NEB) and Illumina TruSeq adapters following the manufacturer's guidelines. Library quality was initially checked by Tapestation D1000 before samples were pooled, the accuracy of pooling was checked on an Illumina Miseq, before sequencing (150 paired‐end) on an Illumina Novaseq 6000 (Illumina, Inc.) to 4‐fold coverage, calculated using as a reference the *M. galloprovincialis* genome, which has a size of 1.7G (Simon, 2022).

**TABLE 1:** Sampling geographical coordinates and number of sequenced individuals in each of the populations used to generate WGS sequencing data in this study.

Samples	Sampling location	Geographical coordinates	*n*° of sequenced individuals	Proposed species
CH	*Carquinez Harbour (USA) – North east pacific*	38.066; −122.231	6	*M. chilensis*
BBH	*Bodega Bay Harbour (USA) – North east pacific*	38.313; −123.051	6	*M. trossulus*
BBC	*Bodega Bay Coastal (USA) – North east pacific*	38.361; −123.070	6	*M. trossulus/M. galloprovincialis*
SBH	*Santa Barbra Harbour (USA) – North east pacific*	34.407; −119.691	6	*M. galloprovincialis*
OA	*Oakland (USA) – North east pacific*	37.805; −122.257	6	*M. galloprovincialis*
CC	*Coliumo, Concepcion (Chile) – South east pacific*	−36.537; −72.958	6	*M. galloprovincialis/M. chilensis*
CV	*Niebla, Valdivia (Chile) – South east pacific*	−39.948; −73.402	6	*M. chilensis*
PM	*Puerto Montt (Chile) – South east pacific*	−41.620; −73.058	6	*M. chilensis*
CSI	*San Isidro (Chile) – South east pacific*	−53.797; −70.992	6	*M. chilensis*
CRS	*Rio Seco (Chile) – South east pacific*	−53.024; −70.780	6	*M. chilensis*
CAF	*Agua Fresca (Chile) – South east pacific*	−53.368; −70.992	6	*M. chilensis*
DDE	*Delta de Ebro, Barcelona (Spain) – Mediterranean*	40.774; 0.763	6	*M. galloprovincialis*
GR	*Eleutherṓn, Nea Peramos (Greece) – Mediterranean*	40.850; 24.321	6	*M. galloprovincialis*
VG	*Playa del Vao, Vigo (Spain) – North east Atlantic*	42.200; −8.789	6	*M. galloprovincialis*
SW	*Kristineberg (Sweden) – North east Atlantic*	58.250; 11.447	6	*M. edulis*
BO	*Bodø (Norway) – North east Atlantic*	67.200; 14.420	6	*M. edulis*
GK	*Kiel (Germany) – Baltic Sea*	54.195; 10.860	6	*M. edulis/M. trossulus*
GA	*Ahrenshoop (Germany) – Baltic Sea*	54.386; 12.427	6	*M. edulis/M. trossulus*
FIN	*Tvärminne (Finland) – Baltic Sea*	59.838; 23.208	6	*M. edulis/M. trossulus*
LF	*Loch Fyne (Scotland) – North east Atlantic*	56.117; −5.240	6	*M. edulis/M. trossulus*
EX	*River Exe, Exmouth (England) – North east Atlantic*	50.362; −3.232	6	*M. edulis*
IC	*Straumsvik (Iceland) – North east Atlantic*	64.020; −22.157	6	*M. edulis*
FAL	*River Fal, Mylor (England) – North east Atlantic*	50.152; −5.024	6	*M. edulis/M. galloprovincialis*

### 2.2 Assessment of population structure and introgression in *Mytilus* spp. using WGS data

To investigate genetic structure and ancestry in the full WGS dataset, an initial filtering was applied in which raw reads were checked for quality and subsequently trimmed and cleaned using fastp v0.19.7 (Chen et al., 2018), removing all reads <100 bp and those with a quality score lower than 20. Clean reads were aligned to the reference genome of a Mediterranean *Mytilus galloprovincialis* mussel (NCBI genome accession JAKGDF000000000), using BWA mem v0.7.17 (Li & Durbin, 2009). Generated files were manipulated with SAMtools v1.3.1 (Li et al., 2009) and BCFtools 1.9 (Li, 2011) and duplicate reads marked using MarkDuplicates (Picard v2.6.0) (http://broadinstitute.github.io/picard). Variants were called using Freebayes v1.3.1 (Garrison & Marth, 2012) from ~26 K contigs larger than >10,000 bp and with mean coverage <20×, estimated BEDtools coverage (Quinlan & Hall, 2010). Coverage of each of the contigs was calculated as the mean coverage in the 138 individuals sequenced in this study and additional 30 individuals provided by collaborators. Those additional individuals were sequenced in combination, and filtered in the same manner, as the samples used in the study, but were not included in subsequent analysis. Resulting SNPs were filtered with GATK v4.0.5.1 (O'Connor & Van Auwera, 2020) using the parameters suggested in the GATK hard filtering germline short variant pipeline (QD < 2.0, FS > 60.0, MQ < 40.0, HaplotypeScore > 13 and MappingQualityRankSum < 13).

Subsequently, variants missing in >50% of individuals within each population were removed and then all samples were merged, before removing SNPs with a minor allele frequency <0.01 in all samples using VCFtools v.0.1.15 (Danecek et al., 2011). Putatively linked loci using window size of 50 Kb, a step size of 10 Kb and an *r* ^2^ threshold of 0.1 were pruned from the dataset using Plink v1.9 indep‐pairwise. We first investigated population structure with a principal component analysis (PCA) (Plink v1.9) and conducted admixture analysis (admixture v1.3) (Alexander & Lange, 2011) among the 23 mussel populations. To determine the best value of *K* clusters (value with lowest cross‐validation (CV) error), we ran admixture testing *K* values between 2 and 10. Data missingness was estimated using the vcfR package in R v4.0.2 (R Core Team, 2021). The final PCA and admixture analysis were ran using SNPs that were genotyped across all populations which were identified using BCFtools v1.9 flag isec (Li, 2011). The results from the population structure and admixture analyses were further applied to determine individual‐level species composition used in the development of the SNP‐array.

### 2.3 Initial quality check, alignment, variant calling and filtering for array development

SNP filtering for the SNP‐array genotyping platform followed the initial filtering steps as described in the section above, but different subsequent steps were applied for filtering: first we removed markers with overall minor allele frequency <0.01, subsequently we kept only markers with monomorphic flanking regions (35 bp in each direction) and, we excluded those which were present in <50% of individuals within each of the 23 populations.

### 2.4 SNP selection for axiom blue mussel array

The set of SNPs with putative monomorphic flanking probes (71‐meter bp) was submitted to Affymetrix ThermoFisher for in silico evaluation. During the quality control (QC) process, each submitted SNP received a design score (p‐convert value) for each 35 bp probe flanking the variant. Based on p‐convert value, probes were classified as ‘Recommended’, ‘Neutral’, ‘Not recommended’, or ‘Not possible’. For subsequent analysis, only variants that scored as ‘Recommended’ for both flanking probes were included.

**FIGURE 2:**
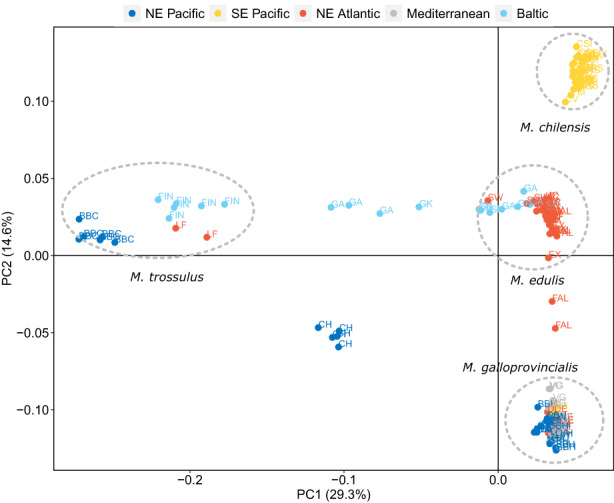
Scatter plots of individual variation in PC 1 and 2 scores resulting from PCA applied to the WGS dataset using SNPs intercepting among all blue mussel individuals from the 23 populations. The proportion of overall variation explained by each PC are given in percentages.

**FIGURE 3:**
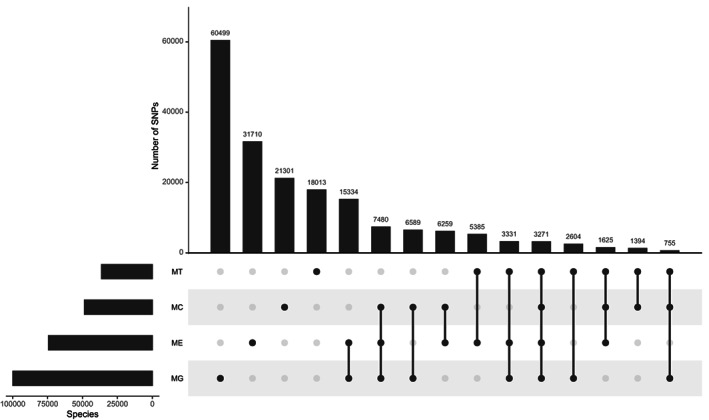
UpSet plot of final set of SNPs with MAF ≥0.02 for each of the species present in the WGS dataset approved by Affymetrix QC and assigned as ‘recommended’ on both flanking probes and their intersection among the four different species group: *M. edulis* (ME), *M. galloprovincialis* (MG), *M. trossulus* (MT), and *M. chilensis* (MC).

Subsequently, SNPs were filtered within each of the four species group (*M. edulis*, *M. galloprovincialis*, *M. trossulus* and *M. chilensis*) by removing markers with a MAF <0.02 within species. Samples were visually assigned to each species group, by analysing sample clusters in the PCA (Figure 2), and markers were classified as either genotyped in all species, three species, two species or species‐exclusive (Figure 3), with those shared on any level among two or more groups being included on the platform (~54 K). To reach the remaining target of 60 K SNPs, markers unique for each species complex (10 K, 2.5 K SNPs from each species) were selected, which were randomly filtered using Plink v1.9 flag –thin‐count (Purcell et al., 2007). In addition, a set of previously published informative SNPs associated with species identification, transmissible cancer, phenotypic sex and population structure in this species complex were provided by collaborators (Table 2).

**TABLE 2:** Information on the previously published set of SNPs added in the platform.

SNP function	Targeted species	n^o^ of SNPs provided	Reference
Species identification	*M. edulis/M. galloprovincialis/M. trossulus*	12	Wilson et al. (2018)
Phenotypic sex	*M. trossulus/M. edulis*	140	Burzyński and Śmietanka (2009), Śmietanka et al. (2010, 2016), Śmietanka and Burzyński (2017)
Transmissible cancer (set 1)	*M. galloprovincinalis/M. edulis*	301	*Alexis Simon pers com unpublished results*
Transmissible cancer (set 2)	*M. galloprovincinalis/M. edulis*	35	Metzger et al. (2016), Vassilenko et al. (2010), Yonemitsu et al. (2019)
Population structure	*M. galloprovincinalis/M. edulis*	113	Hammel et al. (2022), Simon et al. (2021)
Population structure (fluidigm)	*M. trossulus/M. edulis*	113	Mathiesen et al. (2017)
Population structure	*M. edulis/M. chilensis*	96	Bach et al. (2019), Gardner et al. (2016), Larraín et al. (2018), Wenne et al. (2016, 2020), Zbawicka et al. (2014, 2018)

### 2.5 SNP‐array validation

#### 2.5.1 SNP summary statistics

One hundred and twenty‐seven samples from the original discovery population, from which DNA was available from genotyping following the WGS, were genotyped using the Blue Mussel array platform. For each of the markers in the platform, three intensity genotyping clusters were generated using the Axiom Analysis Suite software (v 5.1.1 Thermo Fisher Scientific) to segregate alleles from a single locus.

The standard protocol for marker quality check (QC) using the AxaS software includes selecting thresholds for the following filters: marker call rate (CR), average sample CR and DishQC (DQC). DQC is a quality control metric that measures the amount of overlap between two homozygous peaks created by a subset of non‐polymorphic probes. In this study, we did not identify a subset of non‐polymorphic probes shared by all the genotyped subspecies, most likely due to not identifying polymorphisms present in the flaking regions of the markers during initial filtering, rendering DQC inappropriate as a sample QC metric. For this reason, we used marker CR as a direct sample QC for the array validation, and five different runs were performed in the software using the following approach: firstly, combining all discovery population samples (run‐ALL) and subsequently using samples classified as *M. galloprovinciallis* (run‐GALLO), *M. edulis* (run‐EDU); *M. trossulus* (run‐TROS), and *M. chilensis* (run‐CHIL). These were performed to understand whether a higher proportion of SNPs were called when grouping all species together rather than when analysing each individual species independently. For each of the runs, we analyzed the final number of SNPs called with a marker CR equal or above 90% to 95%. Finally, we accounted for polymorphism among markers within and between species.

#### 2.5.2 Assessment of array performance and applications

To assess the array performance, we compared genotype information within the four species generated by two different methods. The first dataset, obtained from the Axiom Analysis Software, consisted of genotype information generated by the 60 K Blue mussel SNP‐array obtained by filtering markers with a CR of ≥95%, in the four individual species groups (*M. galloprovincialis, M. edulis, M. trossulus* and *M. chilensis*). For the second dataset, we extracted genotype information on the same set of markers for the four species groups from the lcWGS data. Markers and their genotypes were extracted from this dataset using VCFtools v0.1.15.

We explored marker frequency and data missingness in both datasets using Plink (v1.9) (Purcell et al., 2007). We then compared called SNP genotypes in each of the individual species. For this, in each individual, we excluded SNPs with missing genotypes. This was done in both genotype datasets separately (lcWGS and SNP‐array). We then assessed the percentage of genotypes that were equivalently called for the SNPs retained in both datasets. Results are presented as (mean ± standard error) per species.

## 3 RESULTS

### 3.1 Population structure and introgression of *Mytilus* sp generated from lcWGS data

The structure of the 23 *Mytilus* spp populations used to generate the 60 K SNP‐array was assessed by PCA using the 4367 SNPs which intersected among all populations (Figure 2 and Figure S1). The final PCA and admixture analysis were run using 4367 SNPs that were genotyped across all populations. The first two eigenvectors accounted for over 43% of the total variance (Figure S2) with the samples clustering into four primaries, or distinct groups, when analyzed visually (Figure 2). Using PC1 and PC2 it is possible to identify a grouping of the four putative species clusters. Several individuals were located in between the main species clusters (*M. galloprovincialis, M. trossulus* and *M. edulis*), suggesting the presence of hybrids in the sampled populations. The percentage of missing data across this reduced 4367 SNP set was 33% ± 5.

**FIGURE 4:**
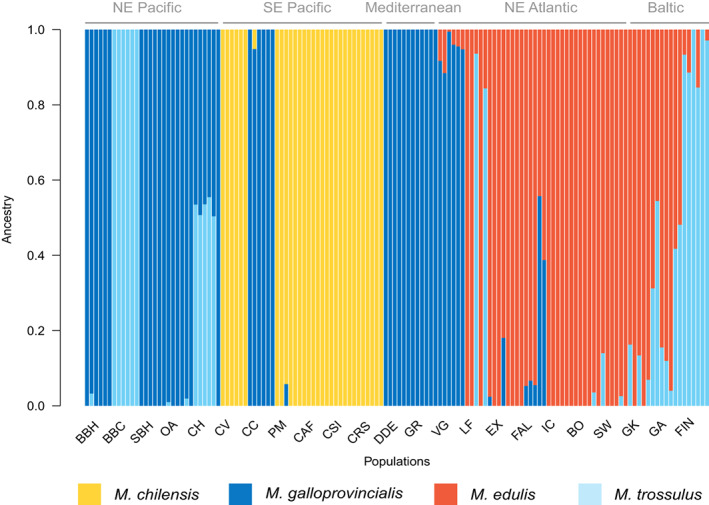
Results of genetic admixture analysis and ancestry inference. In each column the proportions of the different colors show the inferred contribution of the four clusters (Q) to the genomic composition of the given individual. Solid bars represent an individual from a single species background.

Ancestry was explored in blue mussel individuals from each of the 23 populations used for generating lcWGS data in this study with admixture. Admixture coefficients (Q) inference of each individual are presented in Figure 4, where in each column the proportions of the different colours show the inferred contribution of the four clusters to the genomic composition of the given individual. Following evaluation of cross‐validation error for each tested *K* (built‐in the software), the most likely number of identified clusters in our dataset was *K* = 4, corresponding to the four *Mytilus* species we expected to uncover in the lcWGS dataset (i.e. *M. edulis*, *M. galloprovincialis*, *M. trossulus*, and *M. chilensis*). As we have not included any *M. trossulus* samples originating from ‘pure’ populations, as previously described in the literature (e.g. Stuckas et al., 2017), assignments of clusters to specific species in our results are putative, though they agree with previous studies describing the distribution of *Mytilus* species (Araneda et al., 2016; Michalek et al., 2016; Saarman & Pogson, 2015; Simon et al., 2020; Vendrami et al., 2020).

Clustering roughly recapitulated predicted species distribution with the yellow cluster representing *M. chilensis*, the dark blue cluster *M. galloprovincialis*, the red cluster *M. edulis*, and the light blue cluster *M. trossulus* (Figure 4). Populations sampled in the south east Pacific were predominantly assigned to *M. chilensis* apart from one population (CC) which was assigned *M. galloprovincialis*. Samples from the north east Pacific were assigned either to *M. galloprovincialis* or *M. trossulus*, with some hybridization in one of the populations between these two species. Hybrids of all 3 species of the *Mytilus* species complex (*M. edulis*, *M. galloprovincialis* and *M. trossulus*) are present in north Atlantic populations. Baltic populations sampled in this study were assigned to be admixed between the *M. trossulus and M. edulis* clusters, in a pattern common to introgressed populations.

### 3.2 SNP selection and array development

**FIGURE 5:**
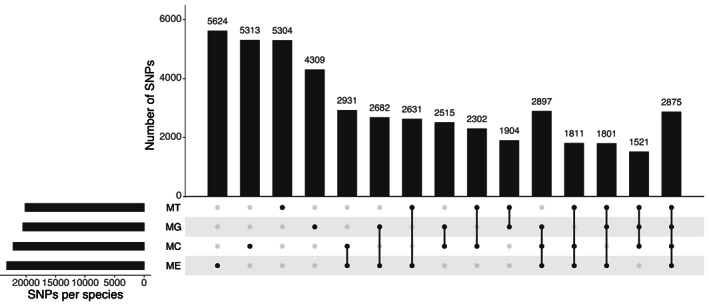
UpSet plot representing probes with a CR of ≥95% retained from the analysis of individual species, *M. edulis* (ME), *M. galloprovincialis* (MG), *M. trossulus* (MT), and *M. chilensis* (MC), generated by the Axiom Analysis Suite Software and their intersection among the four species group.

The alignment of the QC filtered reads against the *Mytilus galloprovincialis* reference genome (genome accession JAKGDF000000000), attainment of bi‐allelic SNPs and post‐alignment QC filters led to the discovery of ~63 million putative polymorphisms. Variants were called from ~26 K contigs larger than >10,000 bp and with <20× coverage across aligned individuals. Biallelic SNPs with a depth coverage of <4 and >10 were discarded, resulting in a dataset of ~31 Million SNPs. Following additional filtering criteria of excluding SNPs with MAF <0.01, those present in less than 50% of genotypes within each population, and those with polymorphic alleles in the 35 bp flanking regions, 1,018,259 SNPs (as 71‐mer nucleotide sequences) were assessed through the Affymetrix ThermoFisher in silico probe scoring. Markers classified as ‘Recommended’ on both flanking probes by ThermoFisher probe QC (233,488 SNPs) were used in subsequent analysis. From this set, SNPs with a MAF <0.02 were filtered from each of the four species groups. The final 60,142 SNP‐array design (Table 3) contained: (i) 54,007 SNPs shared by either all species (3268), three species (13,185) or two species (37,554) (Figure 5), based on species assignment using admixture analysis; (ii) SNPs exclusive for each species (*M. galloprovinciallis* 1339; *M. edulis* 1384; *M. trossulus* 1360 and *M. chilensis* 1361) and (iii) 691 SNPs from the 810 previously identified informative SNPs detailed in Table 2. The final composition of the panel of SNPs is presented in Table 3.

**TABLE 3:** Final design of the Blue Mussel 60 K Array, including information on SNP sequencing origin and species in which the marker is found.

Species	Origin	SNPs (*n*)
Shared among all species	WGS	3268
Shared among three species	WGS	13,185
Shared among two species	WGS	37,554
Unique to *M. galloprovinciallis*	WGS	1339
Unique to *M. edulis*	WGS	1384
Unique to *M. chilensis*	WGS	1361
Unique to *M. trossulus*	WGS	1360
Others	Previous published studies (Table 2)	691
**Total**		**60,142**

### 3.3 SNP‐array validation

#### 3.3.1 SNP summary statistics

In this study, SNPs were retained for genotyping based on marker CR and not sample DQC. In all marker CR filtering scenarios, the number of usable SNPs generated was higher when analysing individual species groups than when analysing all discovery population samples in combination (Table 4). We set the CR of ≥95% as a standard for subsequent analysis, allowing genotyping of individuals in approximately 30% of 60 K markers in all four individual species. With this CR filter, 23,252 markers were retained for genotyping *M. edulis*, 22,165 for *M. chilenesis*, 20,504 for *M. galloprovincialis* and 20,149 for *M. trossulus*.

**TABLE 4:** Number of SNPs applicable for genotyping resulting from multiple call rate (CR) scenarios (from 90% to 96%) using different group of samples: (i) all discovery populations samples and (ii) samples from the different Blue Mussel species used in the array.

	All samples combined	*M. galloprovincialis* (run‐GALLO, *n* = 30)	*M. edulis* (run‐EDU, *n* = 22)	*M. trossulus* (run‐TROS, *n* = 14)	*M. Chilensis* (run‐CHIL, *n* = 23)
	Probes (*n*)	Probes (*n*)	Probes (*n*)	Probes (*n*)	Probes (*n*)
CR (%)					
90	21,542	33,938	31,908	33,058	30,264
91	20,074	27,509	23,252	33,058	30,264
92	18,576	27,509	23,252	33,058	22,165
93	15,570	27,509	23,252	20,149	22,165
94	13,976	20,504	23,252	20,149	22,165
95	12,440	20,504	23,252	20,149	22,165
96	10,837	20,504	13,207	20,149	13,341

We visualized the output of SNPs applicable for genotyping *Mytilus* spp with a CR of ≥95% resulting from the analysis of individual species (Table 4, Figure 5). When combining all groups, 46,420 SNPs were retained as applicable for genotyping, representing 77.2% of SNPs in the array. From this number, 12.1% of the markers were unique for *M. edulis*, 11.4% for both *M. chilensis* and *M. trossulus*, whilst the lowest number of species exclusive markers was observed for *M. galloprovincialis*, with 9.3%. Approximately 6.92% of the SNPs were shared among all species.

**FIGURE 6:**
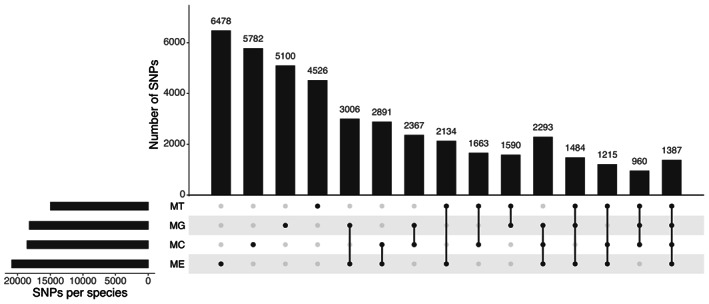
UpSet plot representing polymorphic probes with a CR of ≥95% retained from the analysis of individual species, *M. edulis* (ME), *M. galloprovincialis* (MG), *M. trossulus* (MT), and *M. chilensis* (MC), generated by the Axiom Analysis Suite Software and their intersection among the four species group.

The majority of markers passing a CR threshold ≥95% were polymorphic within species (Table 5). Among the four species, *M. edulis* had the highest number of unique polymorphic markers (15.1%, Figure 6) compared to *M. chilensis* (13.5%), *M. galloprovincialis* (11.9%) and *M. trossulus* (10.6%).

**TABLE 5:** Summary of useable SNPs analysing species individually (*M. edulis*, *M. galloprovincialis*, *M. trossulus* and *M. chilensis*) applying a call rate over 95% filter.

Samples (DNA)	Individuals (*n*)	SNPs category	SNPs (*n*, per category)
*M. galloprovincialis*	30	Total	20,504
		Monomorphic	2317
		Polymorphic	18,187
		Polymorphic unique *to M. galloprovincialis*	5100
*M. edulis*	22	Total	23,252
		Monomorphic	2637
		Polymorphic	20,888
		Polymorphic unique *to M. edulis*	6478
*M. trossulus*	14	Total	20,149
		Monomorphic	5190
		Polymorphic	14,959
		Polymorphic unique *to M. trossulus*	4526
*M. chilensis*	14	Total	22,165
		Monomorphic	3607
		Polymorphic	18,558
		Polymorphic unique *to M. chilensis*	5782

Individual missing genotypes per individual species in the lcWGS dataset was generally high in the four species group: *M. galloprovincialis* (63.11% ± 0.58), *M. edulis* (64.45% ± 0.55), *M. trossulus* (77.24% ± 0.6) and *M. chilensis* (66% ± 0.56) in comparison to the array genotyping dataset: *M. galloprovincialis* (1% ± 0.5), *M. edulis* (2% ± 0.1), *M. trossulus* (0 ± 0) and *M. chilensis* (2% ± 0.1), meaning that across the SNPs commonly called using both technologies, a far greater percentage was successfully called using the array.

For *M. galloprovincialis*, an average of 71.02% ± 4.07 of the genotypes were called equivalently using the two technologies, followed by *M. chilensis*, for which an average of 57.5% ± 1.59 of genotypes were consistently called in both genotyping platforms, *M. edulis* (50.1% ± 3.6) and *M. trossulus* (37.58% ± 3).

## 4 DISCUSSION

In this study, we developed the first high throughput genotyping assay for the four main cultured species in the *Mytilus* genus, a multi‐species medium density 60 K SNP‐array. SNPs were selected from 138 individual mussels, originating from 23 wild, globally‐distributed populations. These successfully called markers were either shared by three species (13,185 SNPs), two species (37,554 SNPs) or all four species (3268 SNPs), or unique to each of the individual groups: *M. galloprovincialis* (1343 SNPs), *M. edulis* (1384 SNPs), *M. trossulus* (1360 SNPs), and *M. chilenses* (1361 SNPs). In addition, 691 SNPs representing markers generated by previous studies were included in the genotyping platform (Table 2). Following the removal of markers with a CR equal or lower than 95%, the final array design retained 23,252 applicable for genotyping *M. edulis* individuals, 22,165 for *M. chilensis*, 20,504 SNPs *M. galloprovincialis*, and 20,149 for *M. trossulus*.

Use of a marker CR of *≥*95% as a threshold instead of DQC, as employed in this study, has been successfully demonstrated in GWAS for different taxa to obtain informative results on population structure, admixture and identification of SNPs significant for breeding purposes, including sheep (Davenport et al., 2020), chicken (Liu et al., 2019) and swine (Cheng et al., 2022; Wijesena et al., 2019). Here we chose to apply this alternative filtering method as samples genotyped in the 60 K blue mussel array consistently received low scores with the DQC parameter (ST1) and were removed from the analysis by the software, indicating the existence of polymorphisms present in the DQC 71 bp flanking probes. This is likely a consequence of the very high polymorphism present in mussel genomes (Romiguier et al., 2014), coupled with the chosen sampling approach of 23 globally distributed mussel populations including four species. Whilst low coverage sequencing allowed the discovery of a large number of SNPs within the samples, it also resulted in patchy data. Choosing to sequence individuals with low coverage might have led to a high missingness, which in turn resulted in a high proportion of the genetic variation being missed during the SNP selection process. This may have also resulted in the removal of accurate data on the presence of SNPs in flanking regions.

In this study, we analyzed the performance of SNPs across different sample sets analyzed in the AxaS software. A higher number of markers were kept when genotyping samples from each species individually (run‐GALLO, run‐EDULIS, run‐TROS, run‐CHIL) than when genotyping all samples combined (run‐ALL). Clusters generated by the Axiom Analysis Software classify the genotypes as either homozygous for one of the two alleles in the probe, or heterozygous. As a consequence of the lcWGS approach chosen to sequence individuals in this study, hemizygous loci, which are missing one of the alleles at a specific position of a marker included on the array, would be incorrectly called by the platform as a missing genotype. Whilst this is a limitation of the genotyping platform, the above mentioned genomic variations are commonly expected to occur in the mussel genome (Gerdol et al., 2020) and might contribute to the high frequency of low quality scores of markers observed in the Blue mussel array. Grouping samples of similar genetic background for analysis reduced the number of genotypes being classified ambiguously (i.e. “Other” and “OTV”), due to distortion of clear genotype clustering that sometimes arose with multispecies sample sets. We appreciate it is not always possible to previously infer the genetic background of samples that will be analyzed using the array. However, our results highlight that grouping samples of similar genetic background improves the number of high‐quality SNPs applicable for genotyping individuals with the array. To increase the number of functional markers applied for genotyping of a sample batch, we propose, when applicable, the employment of an analysis pipeline where samples are first disaggregated by species grouping before subsequently being analyzed across the entire marker set. Nonetheless, the comparable population structure inferred from sample genotypes generated by the array (CR filter of ≥95%) to that generated by the lcWGS dataset, reinforces the suitability of developing a genotyping platform in this species complex.

Subsequently, we analyzed the proportion of polymorphic markers retained within and among the four species groups. A high proportion of the successfully called SNPs on the array are polymorphic within species (*M. edulis* 89.8%, *M. galloprovincialis* 88.7%, *M. chilensis* 83.7%, and *M. trossulus* 74.2%). The proportion of unique polymorphic probes per species was considerable (*M. edulis* 15.1%, *M. galloprovincialis* 11.9%, *M. chilensis* 13.5%, and *M. trossulus* 10.6%). Polymorphism within each of the mussel species in the array was higher in comparison to other multi‐species arrays, such as the ~49.9 K European Pine tree multispecies array, in which approximately one quarter of the converted SNPs was polymorphic in each species in the array (Perry et al., 2020). Nonetheless, in our study, only 960 polymorphic markers were shared among all four mussel species. These observations reassure that although markers are shared between either two, three or four mussel species constituting the array, this marker set is appropriate for investigating population structure and species divergence. Blue mussels, as is the case for other marine bivalve mollusc species, are known to have a highly complex and polymorphic genome (Calcino et al., 2021; Gerdol et al., 2020). Although a high proportion of markers is lost following the QC filtering, a sufficient number (~20 K) is retained for each of the four species when running QC for batches of samples with similar genetic background. Approximately a quarter of these markers are unique per species. These values reassure that the diversity existing within and among species can be captured with the Blue mussel 60 K array. The addition of 691 markers, which have been previously confirmed as species‐diagnostic and relevant for sex determination and identification of cancer‐positive samples (cited in Table 2), further guarantees the applicability of the genomic tool generated in this study as highly valuable for fine scale investigation of genetic structure in these taxa.

The percentage of genotypes called equivalently using both approaches was higher for *M. galloprovincialis* than for the other tested species. Such discrepancies might result from variance in the flanking probes, due to divergence in the genomes of these sister species, interfering in the produced signal or in the physical binding of the genetic material. Furthermore, it is very likely that two additional factors: (i) the higher number of *M. galloprovincialis* individual in the founding and validation populations and (ii) the alignment to a Mediterranean *M. galloprovincialis* genome assembly, may also be contributing to a biased comparison of genotypes in the array. Besides, the high level of missing data across the lcWGS data suggests that this sequencing method was not the optimal approach for SNP selection for this species complex. Especially when taking into consideration the high hemizygous fraction of the mussel genome, the low coverage approach most likely contributed to incorrectly call individuals with a missing locus as a homozygous with a missing loci. Furthermore, these hemizygous regions are highly associated with the dispensable portion of the mussel genome, which, in turn, suffer massive presence and absence variation among individuals. Using a non‐annotated genome assembled at a contig level did not allow the identification of these specific regions, or the selection of SNPs located in core regions. In future, when working with these highly polymorphic species, a different approach, either pool sequencing larger numbers of individuals from each population (Kranis et al., 2013), or whole genome resequencing larger numbers of individuals from each population at higher coverage, will likely yield more useful SNPs to add to the next version of this array platform. In addition, newer versions of the mussel genome are now available, assembled to a higher level. Using these genomes as references would allow the selection of SNPs associated with functional genomic regions, consequently relevant for segregating species/populations more clearly.

We assessed population structure and sample ancestry in the lcWGS dataset using only SNPs genotyped in all populations. With a PCA, we could visually distinguish among four species within the genus *Mytilus*, allocating populations as *M. edulis*, *M. trossulus*, *M. galloprovincialis*, and *M. chilensis*, supported by previous studies of genetic differentiation (Araneda et al., 2016; Fraïsse et al., 2016; Gardner, 1994; Hilbish et al., 2002; Koehn, 1991; Michalek et al., 2016; Saarman & Pogson, 2015; Väinölä & Hvilsom, 1991; Vendrami et al., 2020; Zbawicka et al., 2018). Through this analysis we could observe some striking ancestry patterns in the sampled populations: a complete shift of dominant species composition was observed between sites of Bodega Bay mussel populations (BBC compared to BBH). These sites are positioned less than 20 km apart from each other but differ in their nature: one being a coastal site (BBC) and the other an enclosed harbor (BBH). Hybrids in Carquinez Harbour (CH) were composed of approximately 50:50 genetic contributions from two species, *M. trossulus* and *M. galloprovincialis*. Whilst this pattern is consistent with hybrid genotypes, recent evidence has suggested that ‘dock mussels’ (i.e. mussels inhabiting port environments) in Europe have a similar admixture pattern between *M. galloprovincialis* and *M. edulis* (Simon et al., 2020), and CH populations might be an additional example of such mussels in the north east Pacific. However, further research is required to understand the nature of such admixture patterns in these North West American populations. The Baltic population from Finland (FIN) shows a more advanced introgression pattern between two of the *K* clusters, likely between *M. edulis* and *M. trossulus*. While populations from Kiel and Ahrenshoop (GK, GA) in the contact zone show a mixing of introgressed *M. edulis* and hybrid genotypes, which is in agreement with previous literature (Stuckas et al., 2017). Along the northern Pacific coast, *M. galloprovincialis* is present in sheltered waters, contrasting to its preference in north east Atlantic populations where this species predominantly populates the exposed rocky tidal environments along the coast (Bierne, Borsa, et al., 2003; Hilbish et al., 2002), with the exception of commercial ports (Simon et al., 2020). This observation is potentially consistent with an inverted coupling between local adaption genes and intrinsic species barriers as previously suggested for the inverted genetic‐environment relationship observed with *M. edulis* and *M. trossulus* in the eastern and western Atlantic (Bierne et al., 2011). Finally, mussel populations in Exmouth, southern England, which have previously been described as pure *M. edulis* (Hilbish et al., 2002), are shown to be introgressed with *M. galloprovincialis*, an observation supported by a recent RAD sequencing study (Vendrami et al., 2020). This can be explained by a better efficiency to detect introgression across a semi‐permeable barrier to gene flow with an increased number of markers distributed along a larger portion of the genome, as discussed by Fraïsse et al. (2016). In conclusion, the species distribution patterns revealed by lcWGS data in this study support previous data for the Baltic Sea (Knöbel et al., 2021; Stuckas et al., 2017), Southwest England (Hilbish et al., 2002; Vendrami et al., 2020), Mediterranean (Boukadida et al., 2021; Simon et al., 2021), USA (Saarman & Pogson, 2015) and Chilean (Araneda et al., 2016) populations. Such observations provide a valuable insight into species distributions and admixture in the blue mussel species‐complex.

## 5 CONCLUSIONS AND FUTURE PERSPECTIVES

We have developed the first medium density multi species blue mussel SNP panel, subsequently validating its performance on 127 individuals from 23 globally‐distributed populations. The blue mussel array includes variants present in each of the four main species: *M. edulis*, *M. galloprovincialis*, *M. trossulus*, and *M. chilensis*. This is an open access tool which allows genotyping in a consistent marker set distributed across the *Mytilus* genome. The Blue Mussel 60 K array can be applied for breeding purposes (i.e. parentage assignment, inbreeding level assessment and species/product identification and provenance), contributing to the understanding of genetic architecture of traits of interest, and leading to the optimization of blue mussel aquaculture via genomic selection. Equally, this tool can be used to deepen our understanding of population genetic processes in these taxa. Shedding light on such relevant phenomena can contribute to the development of conservation guidelines and effective management strategies of wild mussel populations as well as those exploited for aquaculture purposes.

## CONFLICT OF INTEREST STATEMENT

The authors declare no conflict of interest for this article.

## Supporting information


Appendix S1.

## Data Availability

Raw sequence reads from the blue mussel samples used for SNP discovery have been deposited in NCBI's Sequence Read Archive (SRA, https://www.ncbi.nlm.nih.gov/sra) under accession number PRJNA932792. The Blue mussel SNP‐array is available for from ThermoFisher (array 551,406 Axiom MytVv1 384S384 Layout, E‐mail: bioinformaticsservices@thermofisher.com).
