# Supplementary material for: SNP discovery and genetic structure in blue mussel species using low coverage sequencing and a medium density 60 K SNP‐array
Source: Evol Appl. 2023 Apr 25;16(5):1044–60. doi: 10.1111/eva.13552 (PMC10197230; doi:10.1111/eva.13552)

# Supplementary material

**ST1** Summary of SNP quality assessment from Axiom Analysis Suite Software using different group of samples: all discovery population samples and samples from the four different Blue Mussel species used in the array. No QC and CR filters have been applied to these datasets.

| **Samples (DNA)** | **(n)** | **SNP conversion** | **SNPs (n, per category)** | **SNPs (Percentage)** | |
| --- | --- | --- | --- | --- | --- |
|  |  |  |  |  | |
| **Discovery populations** | 127 | Other | 50,017 | | 83.1 |
|  |  | OTV | 5,609 | | 9.3 |
|  |  | PolyHighResolution | 2,029 | | 3.3 |
|  |  | NoMinorHom | 1,785 | | 3.0 |
|  |  | MonoHighResolution | 702 | | 1.2 |
|  |  | BestandRecommended | 4,516 | | 7.5 |
|  |  |  |  | |  |
| ***M. galloprovincialis*** | 30 | Other | 38,777 | | 64.5 |
|  |  | OTV | 12,053 | | 20.0 |
|  |  | PolyHighResolution | 3,524 | | 5.9 |
|  |  | NoMinorHom | 2,873 | | 4.8 |
|  |  | MonoHighResolution | 2,915 | | 4.8 |
|  |  | BestandRecommended | 9,312 | | 15.5 |
|  |  |  |  | |  |
| ***M. edulis*** | 21 | Other | 42,590 | | 70.8 |
|  |  | OTV | 10,610 | | 17.6 |
|  |  | PolyHighResolution | 2,291 | | 3.8 |
|  |  | NoMinorHom | 3,138 | | 5.2 |
|  |  | MonoHighResolution | 1,512 | | 2.5 |
|  |  | BestandRecommended | 6,941 | | 11.5 |
|  |  |  |  | |  |
| ***M. trossulus*** | 14 | Other | 43,422 | | 72.2 |
|  |  | OTV | 9,937 | | 16.5 |
|  |  | PolyHighResolution | 1,361 | | 2.3 |
|  |  | NoMinorHom | 3,873 | | 6.4 |
|  |  | MonoHighResolution | 1,549 | | 2.6 |
|  |  | BestandRecommended | 6,783 | | 11.3 |
|  |  |  |  | |  |
| ***M. chilensis*** | 23 | Other | 43,261 | | 71.9 |
|  |  | OTV | 8,540 | | 14.2 |
|  |  | PolyHighResolution | 2,377 | | 5.0 |
|  |  | NoMinorHom | 2,955 | | 4.9 |
|  |  | MonoHighResolution | 3,008 | | 5.0 |
|  |  | BestandRecommended | 8,340 | | 13.9 |
|  |  |  |  | |  |

# Figure S1: Scatter plots of individual variation in PC 1 and 3 scores resulting from PCA. The amount of variation explained by each PC are given in percentages.


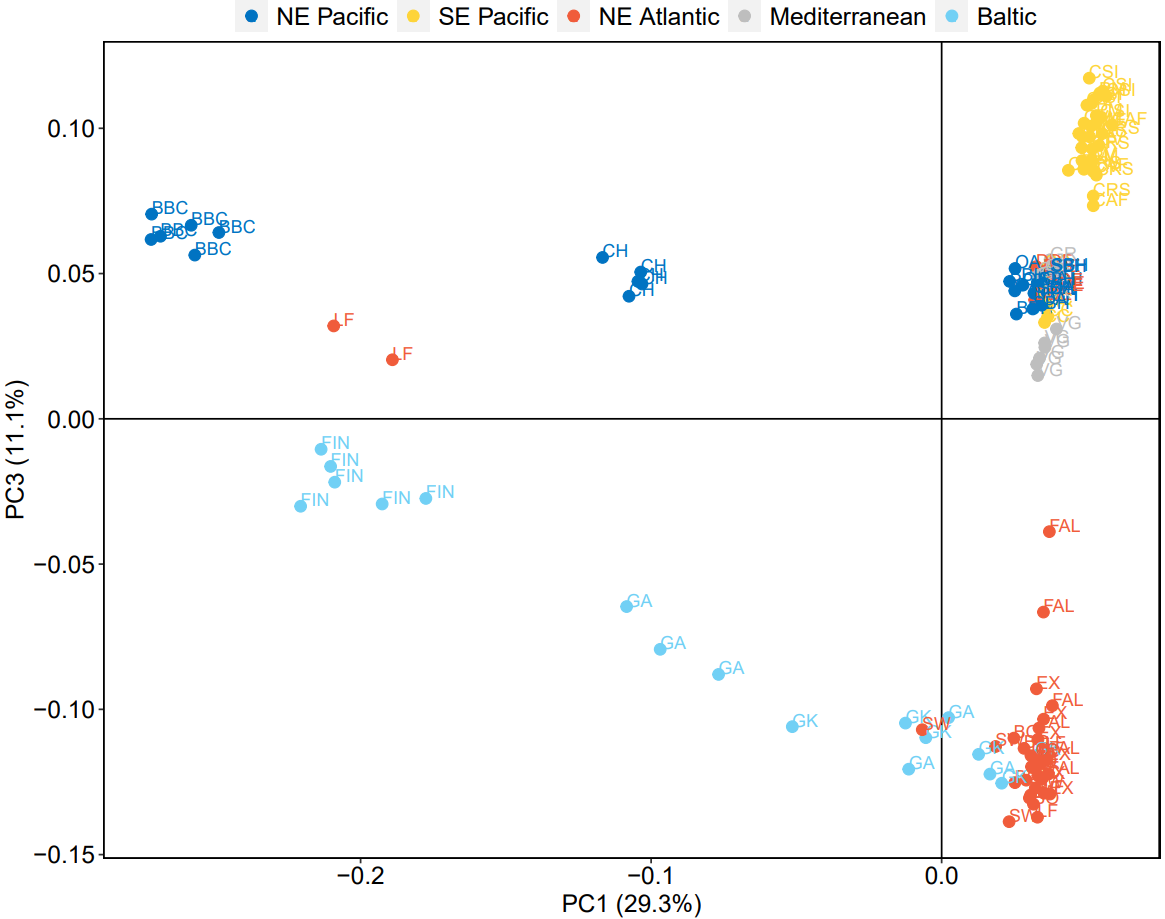


# Figure S2: Bar plot with the percentage of variance explained by each PC (1-20) resulting from the PCA.


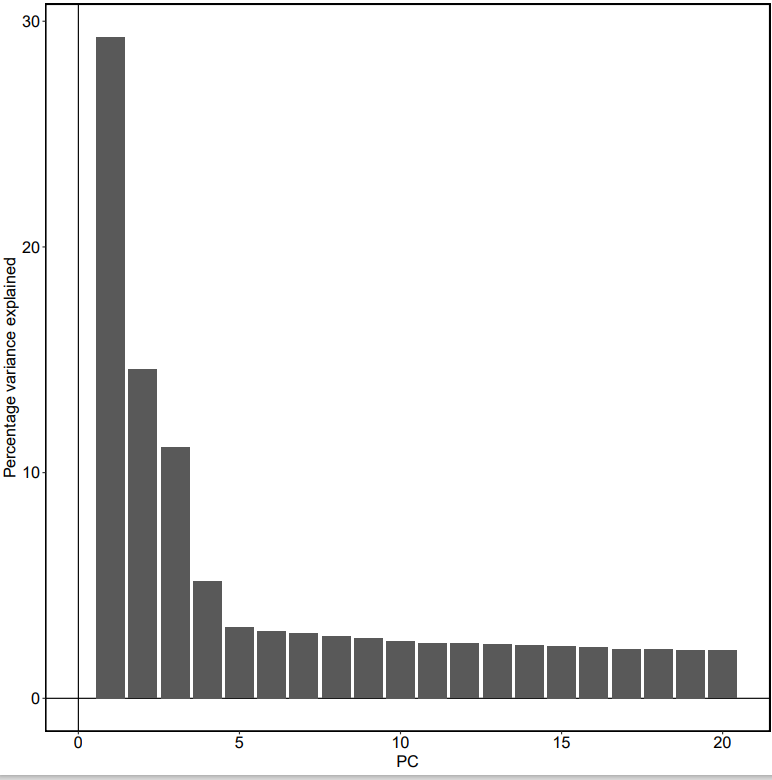

Supplement: Supplementary file 1 — Appendix S1. [file EVA-16-1044-s001.docx]
